# Micro-Elimination: Updated Pathway to Global Elimination of Hepatitis C in Small Communities and Industrial Settings during the COVID 19 Pandemic

**DOI:** 10.3390/jcm10214976

**Published:** 2021-10-27

**Authors:** Anca Elena Butaru, Dan Ionuț Gheonea, Ion Rogoveanu, Mircea Diculescu, Ancuța-Ramona Boicea, Marius Bunescu, Costin-Teodor Streba, Carmen Nicoleta Oancea

**Affiliations:** 1 Department of Infectious Disease, University of Medicine and Pharmacy of Craiova, 200349 Craiova, Romania; anca.butaru@yahoo.com; 2 Department of Gastroenterology, University of Medicine and Pharmacy of Craiova, 200349 Craiova, Romania; dan.gheonea@umfcv.ro (D.I.G.); ion.rogoveanu@umfcv.ro (I.R.); 3 Department of Gastroenterology, “Carol Davila” University of Medicine and Pharmacy, 050474 Bucharest, Romania; mircea.diculescu@gmail.com; 4 Department of Labor Medicine, University of Medicine and Pharmacy of Craiova, 200349 Craiova, Romania; Ancuta.boicea@umfcv.ro (A.-R.B.); marius.bunescu@umfcv.ro (M.B.); 5 Research Center of Gastroenterology and Hepatology of Craiova, 200638 Craiova, Romania; 6 Department of Pulmonology, University of Medicine and Pharmacy of Craiova, 200349 Craiova, Romania; 7 Department of Analytical Chemistry, University of Medicine and Pharmacy of Craiova, 200349 Craiova, Romania; carmen.oancea@umfcv.ro

**Keywords:** hepatitis C viral infection, micro-elimination, screening, target, linkage to care

## Abstract

Background: In response to the goal of the World Health Organisation to eliminate hepatitis C virus infections by 2030, Romania is striving for national elimination. An already successful micro-elimination project was expanded to test-and-treat specific populations and at-risk groups. The aim of this project was to identify the individuals with HCV infection in disadvantaged regions who do not have proper medical care access. Materials and Methods: Our two-arm interventional cross-sectional study used rapid anti-HCV antibody testing on two population groups from the Romanian southwestern region of Oltenia, approached between September 2020 and May 2021. The first group consisted of predominantly over 40 years old individuals, recruited through five family doctors from two medium-sized towns (community lot—CL). We approached a second group, aged 18–65, through 11 medical offices of five large factories in the same region (industry lot, IL). A 12-items questionnaire was given to each participant, to determine risk factors and record demographic data. Eligible patients initiated antiviral therapy using direct-acting antivirals (DAAs). Results: We enrolled 15,383 individuals between all 16 locations. The overall prevalence by antibody testing was 0.77% (119 cases). Of these, 57 subsequently received treatment with DAAs. We identified blood transfusions as a risk factor within the CL. Participants in the IL reported a relatively high risk for the following situations: sharing of personal hygiene belongings with another person, performing previous blood transfusions, dental interventions and previous surgery. Conclusions: In this global context, the use of micro-elimination allows interventions to be faster and more efficient. This is possible by targeting smaller and specific HCV risk groups.

## 1. Introduction

Discovered in 1989, hepatitis C virus (HCV) is a single-stranded enveloped RNA virus that replicates in the hepatocytes [1]. Hepatitis C viral infections can be classified as acute and chronic. The minority of patients will have a spontaneous clearance within 6 months of infection [2]. With a notable propensity to develop a chronic infection, persistent HCV infection can be responsible for liver disease with progressive scarring of the liver (cirrhosis) and hepatocellular carcinoma (HCC) if screening is not followed by treatment [3].

The management of patients with chronic HCV infection has progressed the most in the current biomedical era by introducing the novel drugs in 2013 which target the replication cycle, called direct-acting antivirals (DAAs) [4]. Tolerable, short, exceptionally efficient and with no significant side effects, these drugs have finally allowed all patients, once they are identified and treated, to have over 95% cure rates [5,6].

HCV infection is an important cause of disability and mortality, affecting more than 71 million people around the world, according to the World Health Organisation (WHO). With 1.34 million deaths in 2015, HCV infection is a significant global health challenge [7].

In 2016, WHO authorized the Global Health Sector Strategy which will be focusing on eliminating viral hepatitis by year 2030 [7]. WHO defined the hepatitis elimination goal as lowering the incidence of hepatitis and hepatitis-related deaths by 95% and 65% [8]. In the spring of 2020, the WHO ‘Global Health Sector Strategy on Viral Hepatitis 2016–2021′ updated the plan, recommending screening for all adults. US Preventive Services Task Force (USPSTF) and the Center for Disease Control and Prevention (CDC) advise that all adults aged >18 years should be tested at least once for HCV infection. Pregnant women are also part of the extended screening recommendations [9,10].

The purpose of micro-elimination is to identify the patients from diverse groups throughout screening and immediately facilitate access to treatment. Cascade of care is a useful tool in order to achieve the WHO target to lower the incidence of new HCV infections by 90% and mortality by 65% by 2030 [11].

In order to reach micro-elimination, all infected individuals must be identified through expanded screening. This can only be done by serological testing using non-laboratory-based rapid diagnostic tests to detect the presence of anti-hepatitis C virus antibodies followed by HCV RNA testing for determining actively infected patients and consequent access to HCV treatment, as well as post-cure follow-up. Multiple challenges as stigma, combined with the lack of knowledge regarding risk factors and routes of transmission, while also having no awareness due to region limited medical specialists, can rise psychological walls to overcome.

Appearing at a critical moment in the context of hepatitis elimination, the evolution of global COVID-19 pandemic further revealed the vulnerabilities of the international health care systems.

This project aim was to expand screening and treatment of different specific populations and at-risk groups, before known and unknown cases will develop signs or symptoms of liver disease, but also to compare HCV identification and linkage to care between two different sites in a disadvantaged region.

## 2. Materials and Methods

### 2.1. Overview

We conducted a two-arm interventional cross-sectional study which followed two population groups, between September 2020 and May 2021.

The first group consisted of individuals predominantly over 40 years old. We approached them through five family doctors from two medium-sized towns, representative for marginalized communities from the Romanian southwestern region of Oltenia.

We recruited the second study group, aged 18-65 (and, where applicable, members of their families) through eleven medical offices of five large factories in the same region.

All sixteen doctors involved, be they family doctors or factories medical offices, signed a contract to facilitate our collaboration.

### 2.2. Study Implementation

#### 2.2.1. Premises

Due to the previously identified barriers in connecting HCV patients to care in this kind of disadvantaged regions, we expanded and provided an improved screening and linkage assistance [12]. We defined “disadvantaged regions” as per the accepted European Union’s Parliament definition: “regions in which the per capita brute income does not exceed 75% of the EU median”.

A team consisting of clinicians (gastroenterologists and infectious diseases), pharmacists and nurses has offered counselling, medical care and support, while also supervising and safeguarding the local identification and linkage to care.

#### 2.2.2. Evaluation

**Figure 1:**
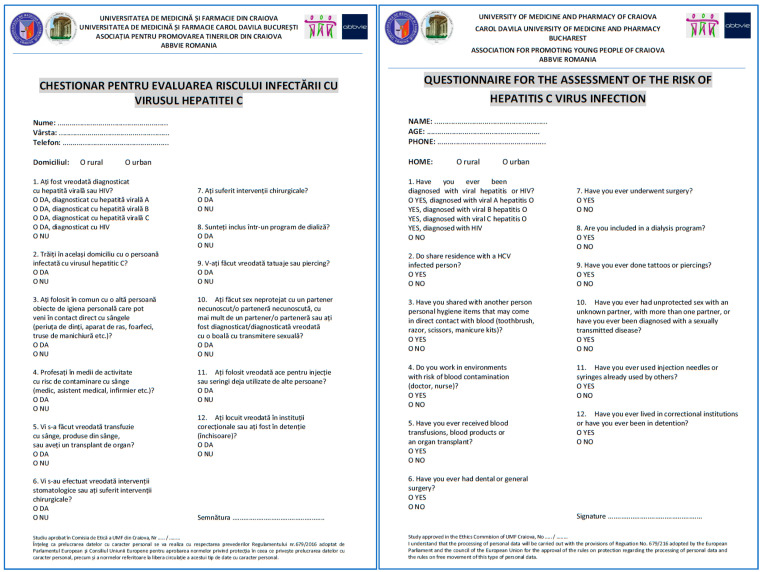
Survey questionnaire (original, in Romanian (**left**) and translated copy in English (**right**)) used to determine risk factors in every patient.

A 12-items questionnaire was completed by all participants in order to properly define risk factors (Figure 1). Design of the questionnaire allowed the identification of several risk factors for HCV infection: cohabitation with HCV positive people, sharing personal hygiene items with another person, working in an environment at risk of blood contamination, having received blood transfusions, dental interventions, surgery, inclusion in a dialysis program, tattoos or piercings, unprotected sex and/or with multiple partners, use of reused syringe needles, staying in correctional institutions or detention.

This approach proved effective for evaluating the awareness regarding HCV. We tracked demographic parameters such as gender, age (at the date of inclusion) and place of origin (urban or rural). The personal data was processed in compliance with the provisions of Regulation no. 679/2016. The phone number was used for further contact, giving the possibility to properly communicate with a potential patient, continue investigations and initiate treatment if needed.

#### 2.2.3. Antibody Testing

All included individuals were tested for the presence of anti-HCV antibodies. The antibody test used for the screening was Anti-HCV TEST WB/S/P (INFO in vitro diagnostic test, Türklab Tibbi Malzemeler San. ve TİC. A.Ş., İzmir, Turkey). With stated 100% specificity and sensitivity (https://www.turklab.com.tr/anti-hcv-test, accessed on 31 August 2021), these qualitative in vitro tests are also single use and disposable. The kits were stored at room temperature and capillary blood samples were taken according to the manufacturer’s guidance by using a sterile, disposable lancet. A result was visible within 5–15 min. This result would be conclusive if a test line appeared next to the control line (which stands for the accuracy of the test), indicating that the test was positive. All these tests and questionnaires were provided by the Association for the Promotion of Youth in Craiova (APT-C).

#### 2.2.4. Care Model

Following identification, the patient received an investigations voucher to ease the complete assessment of the disease status. That consisted in HCV RNA, complete blood count, α-fetoprotein-AFP, albumin, alanine aminotransferase—ALT, aspartate aminotransferase—AST, hepatitis B surface antigen (HbsAg), anti-HBc antibodies, anti-HIV antibodies and international normalized ratio (INR).

Our call centre would take over and keep in touch for more details regarding ongoing investigations. Given the distance, telemedicine greatly helped the collaboration between the medical team and the target community–videoconferences were arranged whenever possible, involving specialists from the University, the patients and their physicians. Questionnaires were processed through a digital on-line platform.

For rural communities lacking the resources, transportation arrangements to the hospital were provided by our team. By doing so, we were able to link the patient to health care, providing all the necessary help. Their progress through the treatment cascade was ensured by the medical team.

Eligible patients were referred to the Research Centre of Gastroenterology and Hepatology within the University of Medicine and Pharmacy of Craiova. Only one appointment was needed, which included Fibromax and abdominal ultrasound. After gathering all this clinical, laboratory and virological data, treatment regimen was decided by the medical team. DAA therapy was available in local pharmacies, which increased the access to treatment. This would also translate into patients being able to pick up their medication quickly.

**Figure 2:**
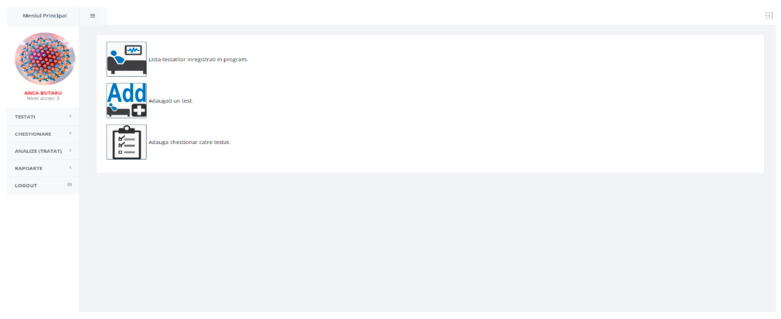
Online platform where each tested patient was recorded.

The University of Medicine and Pharmacy of Craiova provided IT support to develop an online platform (Figure 2). Each tested patient was introduced here, based on the individual questionnaire that was completed. This online platform proved to be of great help in isolating and removing the duplicate data of patients who might have taken the test at two different sites, as well as the questionnaires with an unsatisfactory level of completion. Registration staff ensured proper contact data and constantly made the updates for each patient throughout the project period. The whole team made sure that, if a person had a positive response at the question regarding cohabitation with HCV positive people, each individual would be linked to care.

This project was carried out under auspices of the University of Medicine and Pharmacy of Craiova and the University of Medicine and Pharmacy of Bucharest, in close contact with medical staff from family medicine offices and factories medical offices.

Our regional elimination initiative involved all different stakeholders such as administrative local representatives, healthcare professionals from both primary care and hospital care, researchers and civil society representatives.

### 2.3. Ethical Considerations

The screening project was approved by Ethics Committee of University of Medicine and Pharmacy of Craiova (No. 82/16 September 2020). All patients received standardized consent and GDPR forms in accordance with national and international regulations. We received full support from all participating factories and had their prior approval for conducting the study on their premises.

### 2.4. Statistical Analysis

We performed all statistical calculations using the GraphPad Prism (GraphPad Software, Inc., San Diego, CA 92108, USA) software. Data was presented as median, minimum and maximum values. We used the unpaired t-test to assess differences between means. We assessed data relationship by using the chi square test of independence. We calculated the relative risk (RR) with 95% confidence intervals (95%CI). Descriptive statistics were used when appropriate.

## 3. Results

### 3.1. Distribution of the Study Lots

During the course of the project, we screened 15,383 individuals across 16 participating locations. The study included: 4430 persons from two medium-sized communities through family doctors—community lot (CL) and 10,953 persons at their place of work through occupational medicine offices—industry lot (IL).

All participants responded to the 12-items questionnaire; we eliminated 112 questionnaires from the CL lot and 115 from the IL as being improperly completed; ten (five in each lot) of these belonged to persons with positive antibodies for HCV.

In the CL, females predominated (2580 women, 58.24%). The group included predominantly urban population (2366 participants, 53.41%). Median age was 58 years for males and 55 years for females.

### 3.2. Anti-HCV Seroprevalence

**Figure 3:**
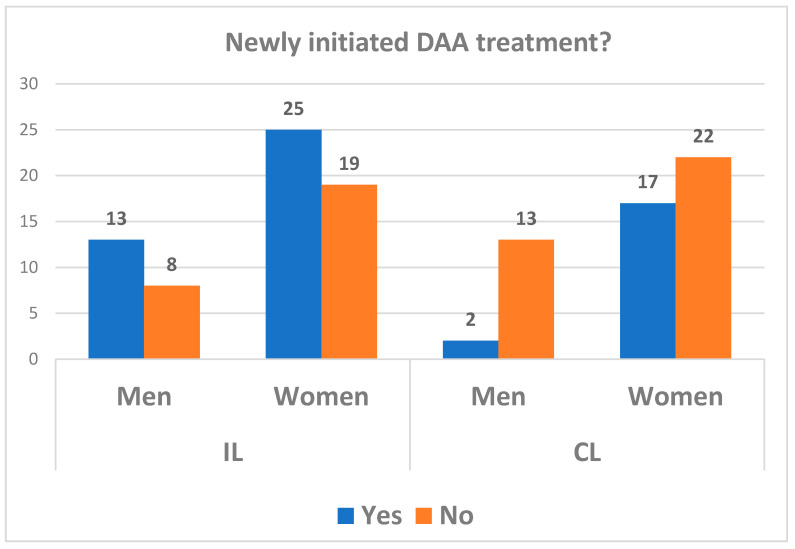
Comparative view of DAA treatments initiated *versus* cases in which antiviral treatment could not be initiated.

The overall presence of anti-HCV-antibodies was 0.77% for the entire lot (119 cases, 54 in the CL and 65 in the IL). Of these, DAA treatment could be initiated in 57 cases (47.8% of the subgroup). A detailed view of these aspects can be found in Figure 3.

We identified anti-HCV antibodies in 54 people (15 men/39 women), 31 from urban areas. Twenty-nine people reported known HCV infections in the questionnaire. We found an incidence of 564.3 cases per 100,000 inhabitants and a prevalence of 1.219% in the CL.

**Figure 4:**
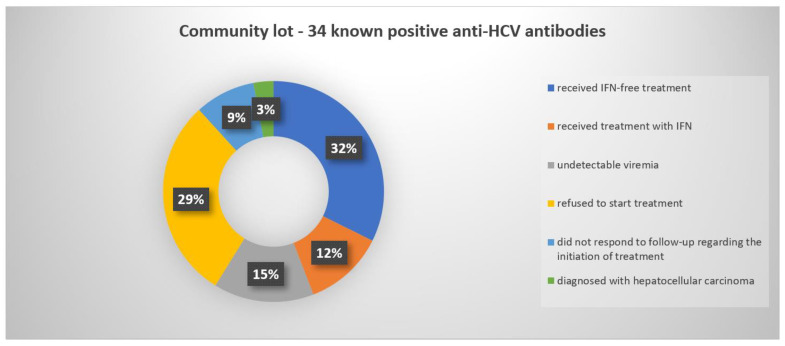
Graphical representation of the status of the 34 persons from the CL with positive anti-HCV antibody results who did not qualify for further treatment.

Linkage attempts were documented for 19 of the 54 patients from the CL, two men and 17 women, and all received treatment (35.18% of HCV-positive persons within this lot). The reasons for not initiating the DAA treatment in the remaining 34 patients can be found in Figure 4.

Males predominated in the IL (6864 men, 62.67%). The group predominantly included urban population (7320 participants, 66.83%). Median age was 42 years for males and 46 years for females.

We identified anti-HCV antibodies in 65 people (21 men and 44 women), 47 from urban areas. Of these, 33 people reported an already known HCV infection in the questionnaire. The industry group revealed an incidence of 292.16 cases per 100,000 inhabitants and a prevalence of 0.593%.

**Figure 5:**
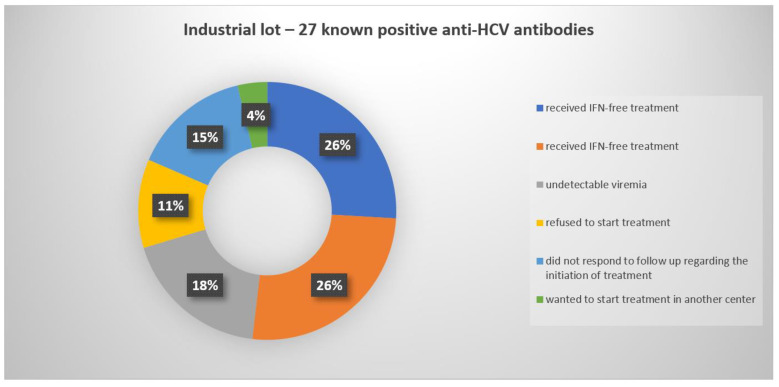
Graphical representation of the status of the 27 persons from the IL with positive anti-HCV antibody results who did not qualify for further treatment.

Of the 65 HCV-positives from the CL, linkage attempts were documented for 38 people (49.23%, 12 men and 26 women, respectively) and all received treatment. For the remaining 27 patients, we detailed the different motives for not initiating antiviral treatment in Figure 5.

We observed statistically significant differences between the two groups, in terms of gender distribution (*p* < 0.001), and also between the average ages in both groups (Student’s *t* test, *p* = 0.0004). We did not notice differences in the distribution by areas of origin, as the urban population predominated in both groups (*p* > 0.05).

We observed a significant relationship between participants’ gender and the presence of anti-HCV antibodies (*p* < 0.05) in both study groups. However, we did not identify a significant relationship between the source environment and the status of anti-HCV antibodies.

### 3.3. Risk Analysis for HCV Infection

Regarding risk factors of acquiring HCV infection, we performed a relative risk analysis for each of the possible etiological factors which resulted from completing the questionnaires. We identified a relatively significant risk regarding blood transfusions (Fisher test *p* < 0.0005; relative risk–RR 3.039 (95% confidence range–95%CI 1.787–4.843)) within the CL. No other risk factor was associated with the presence of anti-HCV antibodies in the CL.

Participants in the IL reported a relatively high risk for the following situations (Fisher test, *p* < 0.05): sharing of personal hygiene with another person (RR 4.561 (95%CI 2.6–7.572)), performing previous blood transfusions (RR 5.365 (95%CI 2.862–9.523)), dental interventions (RR 1.349 (95%CI 1.12–1.536)), surgery (RR 1.335 (95%CI 1.043–1.613)).

We performed a chi-square test of independence which revealed a significant relationship between gender and the presence of anti-HCV antibodies, χ^2^ (1, *N* = 4430) = 4.39, *p* = 0.036 for the CL and χ^2^ (1, *N* = 10,953) = 25.76, *p* < 0.0001 for the IL.

On the other hand, we did not find a significant relationship between place of residence and anti-HCV antibodies; χ^2^ (1, *N* = 4430) = 0.351, *p* = 0.553 for the CL and χ^2^ (1, *N* = 10,953) = 0.884, *p* = 0.346 for the IL.

## 4. Discussion

With approximately 1.75 million new infections a year, hepatitis C virus infection is a global health concern [13]. It is one of the leading causes of chronic liver diseases and liver malignancy all around the world [13]. It also represents an important cause of mortality and morbidity through infected patients in comparison with patients who are not infected or who received treatment [14]. HCV infection became largely curable with the new launched HCV drug regimens without Interferon-α in 2013. They substantially lowered the possibility of long-term complications of the liver disease and is currently the treatment of choice [15,16]. This new era of DAAs determined a sustained virological response (SVR) in more than 95% of the treated patients [17].

The significance of the HCV epidemic made WHO plan the elimination of viral hepatitis by 2030 [8]. HCV global elimination requires expanded screening which leads to identifing undiagnosed cases [18]. The elimination concept targets defined high-risk groups or geographic areas and can help to treat and prevent infections in a more proper way [18].

Hepatitis C prevalence rates between less than 0.5% in western, northern and central European countries [19]. Values between 3 and 8% can be found in the eastern part or outside Europe [20]. It is very important to be aware of the real numbers of hepatitis C infections, thus several projects and studies are being developed to save lives through testing and treatment [21]. A favourable outcome of such micro-elimination programmes would come as a great progress for HCV elimination in Romania.

Our study reports on the screening of 15.383 individuals from two groups, community lot and industry lot. Among them, the overall rate of positive HCV antibody test was 0.77%. HCV antibody prevalence gradually raised with age. This is similar to a study carried out in Egypt, a country with a large reservoir of infection, where the HCV prevalence reached a high percentage in the 50-59 years age group [22]. The present study concluded that more females than males had a positive HCV antibody test.

We also noticed that older females from rural areas were predominant and more susceptible to acquire HCV infections. This can be explained by the fact that elderly people living in those areas do not have access to proper levels of education, awareness and information. It is to be expected that hygiene plays an important role. Also, medical care access is more reduced in rural compared to metropolitan regions. These aspects were noted in a modelling study from United States which showed that targeted and regionally suitable HCV screening and treatment can help rural areas to achieve elimination by 2030 [23]. A similar increasing trend by age together with the positive predominance of female gender was observed in rural areas from Italy [24]. This study illustrates risk factors such as history of blood transfusions or surgery [24].

In this study, a considerably higher risk factor of HCV infection among people with a history of blood transfusions was observed in the industry lot. The literature also suggests that whole blood transfusion and previously received blood products are associated with a risk of HCV infection [25]. There is also evidence that sporadic cases may still occur, however at a lower level because of the taken measures to prevent blood-borne infections in the general population [26,27].

Because of the efforts concerted of all the stakeholders involved, our screening project is a step forward for elimination HCV nationwide. An example of a successful HCV micro-elimination campaign is from Iceland, which is already on time for the WHO goal [28]. Another country close to the WHO goal is the Netherlands, where many micro-elimination projects have helped to close the gap in different targeted populations [29,30,31,32,33,34].

Another key point of our project was easy access to short duration DAA therapy, particularly for remote areas, which will greatly influence the HCV burden of infections. We received positive feedback regarding the treatment, with few and entirely manageable side effects. Comparable findings were reported in a study in a endemic rural area in Taiwan from January to August 2019 where positive patients who received DAA therapy had a high cure rate with few discomforts [35].

In our study, we encountered some limitations due to the improper filling of questionnaires by some of our subjects, leading to a potential source of bias. We carefully reviewed questionnaires through the electronic database and eliminated all suspicious records from our analysis (i.e., questionnaires that answered “yes” on all items). Another aspect would be the reluctance of reporting on some items. Social and cultural factors regarding multiple sexual partners or former prison inmates can be considered a taboo subject, thus leading to severe under-reporting which possibly impaired the correctness of data. On the other hand, reaching some patients for follow-up proved difficult, as some phone numbers were invalid and thus, we could not provide further support.

## 5. Conclusions

This study indicates that screening, comprehensive HCV care and treatment in carefully identified hotspots with deficient access to healthcare system leads to reducing the HCV related issues. Low regional prevalence and incidence in together with universal access to DAAs promote a future HCV elimination success. Few studies have been carried out in healthy individuals from both rural and urban regions and this micro-elimination strategy that we uniquely used should be popularized to benefit all Romanian communities in order to achieve hepatitis C elimination. Essential tools such as awareness and communication campaigns, prevention programmes, testing and treatment programmes, surveillance can lead to successful linkage to care and improved outcomes.

## Data Availability

Anonymized data pertaining to the study can be obtained from the authors upon request.
